# Assessment of Remote Vital Sign Monitoring and Alarms in a Real-World Healthcare at Home Dataset

**DOI:** 10.3390/bioengineering10010037

**Published:** 2022-12-28

**Authors:** Nicole Zahradka, Sophie Geoghan, Hope Watson, Eli Goldberg, Adam Wolfberg, Matt Wilkes

**Affiliations:** 1 Current Health Inc., Boston, MA 02108, USA; 2 Current Health Ltd., Edinburgh EH1 3EG, UK

**Keywords:** remote monitoring, alarm, vital sign, hospital at home, wearable

## Abstract

The importance of vital sign monitoring to detect deterioration increases during healthcare at home. Continuous monitoring with wearables increases assessment frequency but may create information overload for clinicians. The goal of this work was to demonstrate the impact of vital sign observation frequency and alarm settings on alarms in a real-world dataset. Vital signs were collected from 76 patients admitted to healthcare at home programs using the Current Health (CH) platform; its wearable continuously measured respiratory rate (RR), pulse rate (PR), and oxygen saturation (SpO_2_). Total alarms, alarm rate, patient rate, and detection time were calculated for three alarm rulesets to detect changes in SpO_2_, PR, and RR under four vital sign observation frequencies and four window sizes for the alarm algorithms’ median filter. Total alarms ranged from 65 to 3113. The alarm rate and early detection increased with the observation frequency for all alarm rulesets. Median filter windows reduced alarms triggered by normal fluctuations in vital signs without compromising the granularity of time between assessments. Frequent assessments enabled with continuous monitoring support early intervention but need to pair with settings that balance sensitivity, specificity, clinical risk, and provider capacity to respond when a patient is home to minimize clinician burden.

## 1. Introduction

Healthcare and Hospital at Home (HaH) programs have become popular during the COVID-19 pandemic, as hospitals exceeded their inpatient capacity, and the risk associated with in-person care increased [1]. Improvements in technology, such as medical-grade wearables and HIPAA-compliant communication platforms, alongside a clinical imperative to change practice, have made it possible to deliver acute care in remote settings [2]. Monitoring a patient’s overall status, including vital signs, is a standard of care in hospital settings to detect deterioration, facilitate intervention, and avoid adverse events. In a remote setting, a patient is less easily reached, so false alarms are potentially more disruptive and expensive. There is a premium on context and specificity. As we move care out of the brick-and-mortar hospital setting, and into patients’ homes, we must ask the question, is there a direct translation of vital signs and vital sign alarm settings between in-hospital and remote monitoring? Or do we need to develop a new paradigm of alerting that is more suited to this new environment?

One of the first indicators of clinical deterioration is a change in physiological status [3]. In the hospital, a patient’s overall status is assessed with a combination of objective and subjective information collected through vital sign measurements, reported symptoms, and clinical observation [4]. At-risk patients are identified with physiological track and trigger systems (PTTS) that use algorithms to assess vital signs. These algorithms range in sophistication from fixed individual vital sign thresholds, [5,6] to adaptive thresholds, [7] to summarizing multiple vital sign measurements and observations into one metric, such as early warning scores (EWS) [8]. PTTS allow clinicians to standardize assessments and responses to acute illness. The National Early Warning Score (NEWS), for example, is consistently used throughout the National Health Service in the United Kingdom (UK), and the latest iteration (NEWS2) incorporates respiratory rate, oxygen saturation, systolic blood pressure, pulse rate, level of consciousness or new confusion, and temperature. An aggregated score of 5–6 is a medium clinical risk and a key threshold for urgent response, while an aggregated score of 7 is a high clinical risk and escalated to an urgent or emergency response [9].

Assessments of physiological status are driven by the frequency of vital sign measurements, which are typically collected every 8 to 24 h by the clinical staff in a general unit [10]. Large gaps in time between vital sign measurements allow for clinical deteriorations to go undetected and may result in adverse clinical outcomes [3]. The low frequency of assessments may be compounded by incomplete sets of vital signs resulting from clinician selection of vital signs measured [11]. A previous study reported that as little as 21% of the 229 vital sign-related interactions between nurse and patient involved a full set of vital sign measurements [11]. Intermittent vital sign measurements generate sporadic information that is not consistently assessed, recorded, interpreted, or actioned [4,12,13]. Nurses agreed that continuous vital sign monitoring would enhance patient safety in the general ward [14,15]. When vital signs are measured by a nurse, an artifact may be introduced to the reading due to patient engagement; during manual observations, patients typically wake, sit upright, and remain still, resulting in vital sign measurements that may not be representative of their physiological status during activities of daily living.

The likelihood of early identification of a clinically significant change in patient status increases as vital signs are measured more frequently [16]. Hospital units where patients are likely to be medically unstable, such as intensive care, high dependency, and post-anesthesia care units, use continuous monitoring systems that often include invasive metrics, such as arterial or central venous blood pressure, with alarm settings that are highly sensitive to acute changes. The number of alarms reported for a patient ranged from 6.5–45.5 per hour on an ICU [17,18] and these units typically have a 1:1 or 1:2 nurse-to-patient ratio. A high nurse-to-patient ratio is required to be able to respond to alarms and intervene appropriately when alarms have high sensitivity. Patients admitted to these units spend most of their time stationary, as mobility is typically limited, either by pathology or equipment. Continuous monitoring is therefore comparably straightforward, as limited motion means the quality of continuous vital sign measurements is better, while tethered equipment is not as much of a hindrance as in other hospital units, where patients are encouraged to move more frequently as part of recovery [19].

Advances in wearable technology have created an opportunity for continuous monitoring to exist outside of high acuity [20] and hospital settings. This brings benefits but can also result in information overload [21]. New equipment, alarm settings, and the interpretation of increased vital sign measurements may end up being perceived as more of a burden than a benefit without proper training. Clinicians may not always recognize deterioration, but successful incorporation of continuous vital sign monitoring into decision making requires an understanding of its strengths and limitations—it is a new paradigm of monitoring, not simply an increase in the rate of intermittent observations [14]. Pairing alarms and EWSs with continuous monitoring may help clinicians recognize deterioration, but the tradeoff is an increased likelihood of false alerting and potential alarm fatigue, especially if alarm settings are not selected with the new context in mind. Actionable alarms are already a low percent (20–36%) of the total number of alarms triggered in adult ward settings [22]. The percent of unactionable alarms is likely to be higher when alarm settings traditionally used in hospitals are applied to vital sign measurements that are collected in a less controlled environment and do not account for factors such as physiological variability, activity, adherence to wearing the device, and measurement accuracy. Vital signs collected through wearables are susceptible to motion artifacts, which decrease the signal-to-noise ratio and can impact accuracy [23].

When implementing vital sign monitoring in HaH programs, clinicians are responsible for decisions that have tradeoffs between the risk of delayed/missed deterioration and alarm fatigue. A high frequency of vital sign measurements without appropriate alarm settings leads to a lot of data without actionable information (or too much actionable information), while a low frequency of vital sign measurements, regardless of the alarm settings, leads to late or missed deterioration. This balance becomes even more important during remote monitoring because care teams rely on patients to wear their devices correctly, and the subjective information collected through clinical observation is not as readily attainable as when a patient is in a hospital room. There is a limited amount of evidence on vital sign collection frequency and alarm recommendations for use in a HaH program. The purpose of this work is to evaluate the effects of vital sign observation rates and alarm settings on alarms using a method to simulate alarms in a real-world dataset collected remotely in a clinical setting. Alarm metrics under different simulated vital sign observation rates and alarm setting conditions demonstrate what would be observed and highlight the impact of the decisions a clinician makes when setting these parameters.

## 2. Materials and Methods

### 2.1. Current Health (CH) Platform

Current Health is a system that supports the remote delivery of care to patients in programs such as healthcare at home. The FDA 510(k)-cleared platform includes an upper-arm wearable that continuously monitors pulse rate (PR), oxygen saturation (SpO_2_), and respiratory rate (RR). Additional parameters, such as “Motion Level,” “Perfusion Quality,” and “Wearable-On-Arm,” are derived from the sensor signals in the CH wearable to provide context to the healthcare provider. Pairing vital signs (PR, SpO_2_, and RR) with movement and patient adherence to wearing the CH wearable offers some compensation for the clinical observation that is unavailable in a remote setting. These parameters are also used to improve the quality of vital sign observations by excluding those collected during unstable conditions, such as high levels of patient movement or incorrect wear. When using the Current Health Generation 2 (Gen2) wearable sensor, the CH platform outputs observations for PR, SpO_2_, and RR at rates of 30, 30, and 15 observations per minute, respectively. This generates 43,200 PR and SpO_2_ observations, and 21,600 RR observations every 24 h per patient. The CH platform uses a rolling median with an aggregation window (*AW*), the window of time the median was calculated over, and a minimum number of observations within *AW* to reduce variability in continuously collected vital sign observations [24]. The minimum number of observations was set to 20% of the expected number of observations for *AW*, determined by the observation rate (Table A1). The CH platform also integrates with peripheral devices to collect blood pressure, axillary temperature, lung function measures, weight, and patient-reported outcomes delivery via tablet; however, these data were excluded for dataset completeness, as not all HaH programs used peripheral devices.

### 2.2. HaH Program Dataset

Data from six HaH programs using the CH platform were screened for eligible patients. Inclusion criteria were HaH admission > 24 h, use of Gen2 wearable, and Gen2 wear time > 24 h. Exclusion criteria were multiple CH platform admissions, and test patients identified by “test” in first/last name, invalid health service ID, or invalid age (<20 or >130 years).

Seventy-six HaH patients with a variety of conditions who were admitted to the CH platform between April 21 and May 15 and discharged before 31 May 2021, were available to be included in the dataset. Patient demographics were limited to data available on the CH platform. The patients were 60 ± 16 years old (*n* = 42), 14 male and 11 female. The reported ethnicities were “Caucasian”: 17; “African American”: 4; “Southeast Asian”: 1; and “Other”: 3. Gender and ethnicity were not reported in 51 patients. Each patient’s dataset was composed of CH platform timestamps, such as CH platform admission timestamp (*T_a_*) and CH platform discharge timestamp (*T_d_*); PR, SpO_2_, and RR values; and PR, SpO_2_, and RR observation timestamps, such as initial vital sign observation timestamp (*T_vs_i_*) and final vital sign observation timestamp (*T_vs_f_*).

### 2.3. Vital Sign Observations

The vital sign observation dataset (*VS_OD_*) was smoothed with a rolling median (*AW* = 5 min, Table A1), akin to the platform’s deployment in clinical practice. *VS_SD_* was used to create datasets with observations every 15 min (*VS_15_*), 1 h (*VS_1_*) [25], 4 h (*VS_4_*) [26,27], and 12 h (*VS_12_*) [9] to simulate vital sign observation frequencies that are common in hospital settings across the acuity spectrum (from the operating room and intensive care to general wards and ambulatory clinics). Observation time was factored into being as representative of in-person measures as possible. To simulate in-person measures, observations from *VS_SD_* were downsampled to every 12 h, 4 h, 1 h, and 15 min starting at 6am (Figure S1). The nearest observation was used when data were missing from the minute mark up to 30 min before or after the minute mark. For example, a vital sign observation from 6:05 am would be used for the 6am downsample time in the absence of any observations between 5:55 and 6:04 am.

### 2.4. Vital Sign Alarms

The physiological track and trigger component of the Current Health platform is designed to support algorithm customization. The trigger-based vital sign alarms are driven by a ruleset that contains vital sign rules; rules include vital sign threshold(s), logic statements, and an aggregation window. The ruleset can be tailored to meet the needs of the use case, taking into account the patient population, planned interventions, expected clinical course, physical distance and response times, and staffing capacity, and it is established prior to CH platform deployment. Rulesets may then be modified based on subsequent experience; vital sign thresholds may be modified at the patient level. The rulesets used in this evaluation were created to identify changes in SpO_2_ (hypoxia), changes in PR (tachycardia, bradycardia), and changes in RR (tachypnea, bradypnea). These changes are indicators of deterioration in a broad range of patients.

Vital sign alarms are affected by vital sign observation frequency, and alarm rules, thresholds, and aggregation windows. To compare the alarm output of different alarm parameters with each other and with in-person clinical monitoring (vital sign observation conditions), we replicated the alarm system so that previously collected patient data could be passed through retroactively (Figure S2). Three vital sign alarm rulesets were evaluated: a subset of NEWS2 rules (*A_1_*) [9], individual vital sign rules (*A_2_*) [5,6], and one primarily designed with combination rules (*A_3_*). Table 1 outlines the rules, including vital sign thresholds and combination rules, for each of the three rulesets. Each vital sign alarm ruleset was tested on *VS_15_*, *VS_1_*, *VS_4_*, and *VS_12_* with an aggregation window set to 0. As described earlier, an aggregation window indicated how many data points to use when smoothing the vital sign dataset (*VS_OD_*) (Figure S1). Four aggregation window conditions were tested for each vital sign alarm ruleset: 5 min (*VS_SD_*), 15 min (*AW_15_*), 1 h (*AW_1_*), and 4 h (*AW_4_*). A timestamp log was generated for each test condition when the vital sign dataset was run through an alarm simulator (Python Software Foundation. Python Language Reference, version 3.8. Available at http://www.python.org (accessed on 10 November 2021) to indicate when an alarm ruleset condition was met. Timestamps were generated for each rule and then grouped for the vital sign alarm ruleset. Where the rulesets included logic statements with multiple vital signs, an overlap window of 30 s was used, so the vital signs needed to have breached their thresholds within 30 s of one another to trigger the alarm.

### 2.5. Data Analysis

A patient’s length of stay (*LoS*) in the HaH program was defined as

(1) $$LoS=T_{d}-T_{a}$$

where *T_a_* and *T_d_* were the timestamps of CH platform admission and discharge, respectively. A patient’s length of wear (*L_w_*) was defined as

(2) $$L_{w}=T_{vs\_f}-T_{vs\_i}$$

where *T_vs_i_* and *T_vs_f_* were the timestamps of the first and last vital sign observations, respectively.

To calculate adherence for each patient, the *LoS* was divided into 15-min windows. A window with at least 1 wearable-on-arm “fact” was counted as an adherent window. The window of wear was the difference in time between the first and last consecutive adherent windows. Window without wear was the difference in time between the first and last consecutive non-adherent windows. A count of the windows of wear was used to calculate the number of times a patient removed the CH wearable during their *LoS*. Wearable adherence was the number of adherent windows per *LoS*. Daily adherence (*A_D_*) was the number of adherent windows per day (24 h). The amount of data each patient contributed to the dataset was defined as the number of adherent windows per patient divided by the total number of adherent windows in the dataset. The number of hours monitored was defined as the sum of the windows of wear for all patients.

To assess alarm conditions, the patient’s *LoS* was divided into 4-h assessment windows similar to the assessment frequency on a general medical/surgical ward [26,27]. An assessment window was included for adherence ≥ 50% (≥8 adherent 15-min windows). A positive alarm window (*W_AP_*) was an assessment window with at least one alarm trigger. The log of timestamps generated by the alarm simulator (described earlier) was grouped into assessment windows. Each assessment window included an initial trigger timestamp (*T_i_*), final trigger timestamp (*T_f_*), and a count of triggers. Total events were defined as the sum of *W_AP_*. The patient rate was defined as the percentage of patients with at least one *W_AP_*. The alarm rate was the average *W_AP_* per patient per day. Early detection time (EDT) was the time difference (in hours) between *T_i_* and the end of the assessment window.

Descriptive metrics of the dataset, CH wearable use, length of stay, vital signs, and alarms were summarized as mean ± SD when normally distributed and median (IQR) when not. Normality was tested with visual inspection and the Shapiro–Wilk test. Vital signs during patient admissions were calculated using the *VS_SD_* dataset. Alarm metrics (*W_AP_,* patient rate, alarm rate, and *EDT*) were compared between alarm rulesets (*A_1_*, *A_2_*, *A_3_*), vital sign observation rates (*VS_12_*, *VS_4_*, *VS_1_*, *VS_15_*), and aggregation windows (*AW_4_*, *AW_1_*, *AW_15_*, *VS_SD_*). Alarm metrics were also compared between vital sign observation rate vs. aggregation window for the following conditions: *VS_4_* vs. *AW_4_*, *VS_1_* vs. *AW_1_*, *VS_15_* vs. *AW_15_*. Patient rate and *W_AP_* were compared between vital sign alarm threshold values for the following vital signs: SpO_2_ (91% vs. 92%); RR (20 breaths/min vs. 25 breaths/min, 10 breaths/min vs. 8 breaths/min); PR (100 beats/min vs. 131 beats/min; 60 beats/min vs. 45 beats/min vs. 40 beats/min). Analyses were performed using GraphPad Prism 9 Version 9.3.1. (GraphPad Software, San Diego, CA, USA).

## 3. Results

The dataset included 76 HaH patients with a median *LoS* of 10 (9.7) days and 12,869 h of monitoring with Current Health. Each patient’s admission contributed a median of 1.07% of data to the dataset, and contribution ranged from 0.05–3.74% of data, depending on the patient’s *LoS* and adherence to wearing the wearable. Respiratory rates from all 76 patients were included in the vital sign dataset. Pulse rate and oxygen saturation were included from 75 patients for 3 of the 4 sampling conditions (*VS_15_*, *VS_1_*, *VS_4_*) and included from 74 patients for *VS_12_*. Missing vital sign data (exclusion from the vital sign dataset) was a result of not meeting the minimum number of data points for the aggregation window criteria. For vital sign observation data, this minimum number of data points was set to 1, as the downsampled data had 1 data point per timestamp. Alarm conditions were evaluated for 3270 4-h assessment windows.

**Figure 1:**
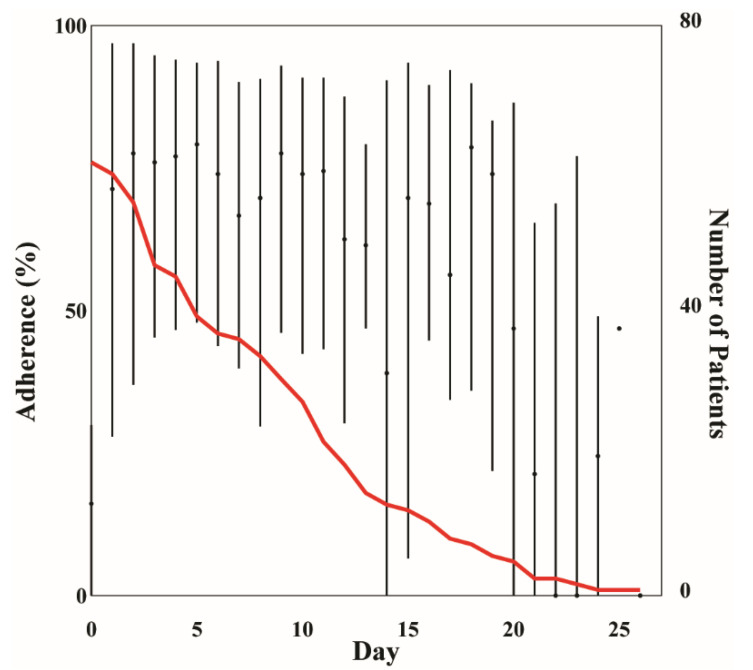
Median (IQR) adherence per day during healthcare at home (HaH) admission. The red line indicates the number of patients enrolled in HaH per day.

Vital sign alarms were triggered by vital sign observations collected when the CH wearable was worn during a patient’s healthcare at home admission. The median CH wearable *L_w_* was 7 (9.2) days. Median adherence during admission was 64.6 (63.5)%, and ranged between 0 and 100% (Figure 1). During the first 10 days (median *LoS*), median adherence ranged between 65 and 80%, except on Day 0 (*A_D_* = 16.15%). Low adherence on Day 0 was potentially attributed to not having the wearable to wear for the full day. The wearable was removed approximately twice per day with a median window of wear of 2 (13.25) h. The median window without wear was 1 h, with a maximum window without wear of 8 days. This 8-day window without wear (max window without wear) was the time between a patient’s last observation recorded and discharge from the CH platform. This window of time either resulted from a patient not wearing the wearable as prescribed or a delay in discharging the patient from the CH platform after the remote monitoring period ended.

### 3.1. Alarms

Alarm metrics are reported in Table 2 for alarm rulesets (*A_1_*, *A_2_*, *A_3_*), observation rates (*VS_15_*, *VS_1_*, *VS_4_*, *VS_12_*), and alarm aggregation windows (*VS_SD_*, *AW_15_*, *AW_1_*, *AW_4_*). The *A_2_* ruleset (rules used only individual vital signs) was triggered most often (*W_AP_*), had the largest *EDT*, patient rate, and alarm rate, while the *A_3_* ruleset (3 of 4 rules used multiple vital signs) was triggered least often and had the smallest *EDT*, patient rate, and alarm rate for all observation rate and alarm aggregation window conditions except for *EDT* of *VS_12_* and *VS_4_* which were zero for both alarm rulesets. The *A_2_* ruleset had the most *W_AP_* when the conditions were compared across the rulesets.

#### 3.1.1. Vital Sign Observation Rate

Comparisons of alarm metrics between vital sign observation rates showed that *A_1_* and *A_3_* had a similar increase in patient rate of ~35% between *VS_12_* and *VS_15_*; however, patient rate of *A_1_* was two times greater than *A_3_* at *VS_12_*. Although the patient rate of *A_2_* only increased by ~7% between *VS_12_* and *VS_15_*, the patient rate at *VS_12_* was high (93%) and reached 100% at *VS_15_*; all 76 patients had at least one *W_AP._* Alarm rate of *A_3_* only increased by 0.9 alarms per patient per day between *VS_12_* and *VS_15_* even though there were 48 times more observations at *VS_15_* compared to *VS_12_*. *W_AP_* and *EDT* increased with increased vital sign observation rates for all alarm rulesets.

#### 3.1.2. Alarm Aggregation Window

In contrast, increased aggregation windows were associated with decreased *W_AP_*, patient rate, and alarm rate for all alarm rulesets. For patient rate and alarm rate, the changes between aggregation window conditions were similar magnitudes to those between vital sign observation rate conditions. For example, the patient rate decreased by 30.3% and 32.9% between *AW_15_* and *AW_4_* for *A_1_* and *A_3_*, respectively, and the alarm rate decreased by 0.6 alarms per patient per day between the two conditions for *A_3_*. *EDT* increased with increased aggregation windows between *AW_15_*, *AW_1_*, and *AW_4_*. For two aggregation window conditions (*AW_1_* and *AW_4_*) of *A_2_*, *EDT* was 4 h, indicating that the alarm ruleset criteria were met at the start of the assessment window.

Comparisons of alarm metrics between similar timeframes of vital sign observation rate and aggregation window showed that most aggregation window conditions had lower *W_AP_*, patient rate, and alarm rate, and greater *EDT*s than their vital sign observation rate counterparts. For example, *A_3_* at *AW_1_* (1-h aggregation window) had a lower patient rate (*VS_1_* = 61.8%, *AW_1_* = 48.7%) and alarm rates (*VS_1_* = 0.6 alarms per patient per day, *AW_1_* = 0.5 alarms per patient per day) and a median *EDT* 1.25 h earlier than *A_3_* at *VS_1_* (1-h observation rate).

#### 3.1.3. Alarm Rule

The vital sign with the highest *W_AP_* was RR, followed by SpO_2_ (Table S1). Rules using these individual vital signs triggered over 1000 *W_AP_* in *A_1_* and *A_2_* rulesets and produced at least 1 *W_AP_* in the patient dataset that ranged from 84.2–100% for the conditions that follow. The conditions in which this occurred for *A_1_* were *VS_15_*, *VS_SD_*, and *AW_15_* (*A_1_-02*: RR ≥ 25); and *VS_15_* and *VS_SD_* (*A_1_-03*: SpO_2_ ≤ 91). For the *A_2_* ruleset, the conditions it occurred in for SpO_2_ < 92 were *VS_15_*, *VS_SD_*, and *AW_15_* and all conditions except *VS_12_* for RR > 20. There were seven *A_1_* rules and one *A_3_* rule that were not triggered in the alarm evaluation. Alarm rules *A_1_-04, A_1_-09,* and *A_1_-10* were not triggered in *VS_12_*. *A_1_-10* was not triggered in *VS_15_*, *VS_1_*, *VS_4,_ AW_15,_ AW_1_*, and *AW_4_*. *A_1_-01* was not triggered in the aggregation window conditions, except for *VS_SD_*. *A_1_-04* was not triggered in *AW_1_*, and *AW_4_*. *AW_4_* did not include triggers from *A_1_-05* or *A_1_-08*. There were no *W_AP_* in any of the observation rate or aggregation window conditions for *A_1_-06*. There were no *W_AP_* for *A_3_-03* (RR < 10 and SpO_2_ < 90) during *VS_12_*, *VS_4_*, *AW_15,_ AW_1_*, and *AW_4_*.

#### 3.1.4. Alarm Threshold

**Figure 2:**
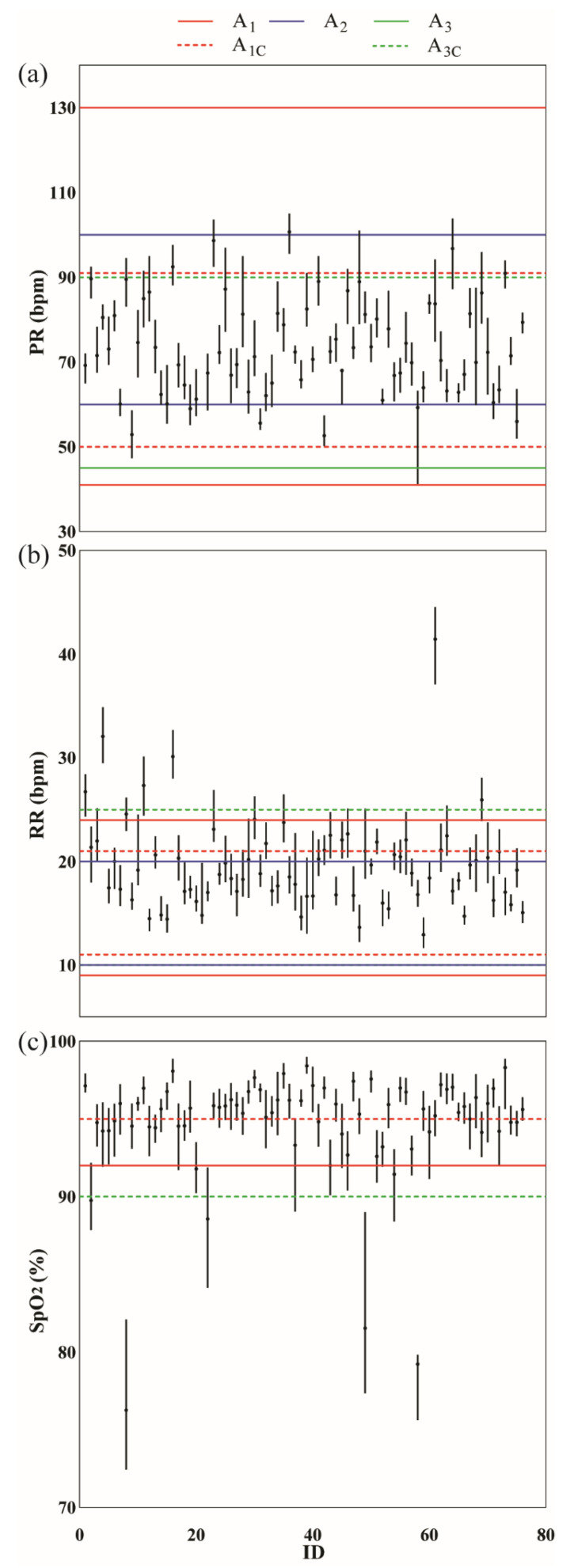
Median (IQR) vital signs for each patient in the healthcare at home dataset (*VS_SD_*). (**a**) Pulse rate (PR); (**b**) Respiratory rate (RR); (**c**) Oxygen saturation (SpO_2_). The horizontal lines indicate the threshold values set for each alarm ruleset. Solid horizontal lines are thresholds used in single vital sign rules: *A_1_* (purple), *A_2_* (cyan), *A_3_* (magenta). Dashed horizontal lines are thresholds used in combination rules: A_1C_ (cyan), A_3C_ (blue).

Vital sign alarm threshold values affected *W_AP_* and patient rates. Threshold values closer to the typical vital sign range (Figure 2) were associated with increased *W_AP_* and patient rates. The median PR was 72.9 (19.4) beats/min, median RR was 19.3 (6.4) breaths/min, and median SpO_2_ was 95.4 (3.6)% during patient admission to healthcare at home programs. An SpO_2_ threshold value to detect hypoxia of 92% compared to 91% increased the patient rate by a median of 8.6 (3.3)% across alarm conditions. There were 193 (106) more *W_AP_* when using an SpO_2_ threshold of 92% instead of 91%, an increase of 33.0 (11.8)%. When comparing RR thresholds to detect tachypnea, a threshold of 20 breaths/min increased patient rate by a median of 22.4 (24.0)% and *W_AP_* by 1096 (165) windows, an increase of 121.0 (93.9)%, compared to a threshold value of 25 breaths/min. To detect bradypnea (low RR), a threshold value of 10 breaths/min increased the patient rate by 9.9 (12.2)%, which was 8 (9) more patients than when the RR threshold was set to 8 breaths/min and had 19 (37) more *W_AP_*. A PR threshold to detect tachycardia of 100 beats/min compared to 131 beats/min increased the patient rate by a median of 46.1 (14.5)% and *W_AP_* by a median of 454 (356) across conditions. A PR threshold to detect bradycardia of 60 beats/min increased the patient rate by a median of 48.0 (9.5)% and 54.6 (12.5)% compared to thresholds of 45 beats/min and 40 beats/min, respectively. There were 489 (273) and 450 (283) more *W_AP_* for a threshold of 60 beats/min across conditions vs. 45 beats/min and 40 beats/min, respectively.

## 4. Discussion

A systematic evaluation of the impact that the selection of vital sign observation rate, alarm rule, alarm aggregation window, and alarm threshold have on vital sign alarms was conducted on a real-world vital sign dataset. Simulating alarm conditions gave us important metrics about the alarm rate, patient rate, and detection time for our patient population, which would have been impossible to gather without the alarm simulation. The vital sign dataset was collected with the Current Health wearable, which continuously recorded oxygen saturation, respiratory rate, and pulse rate from patients admitted to HaH programs in the US and UK. Patient acuity was akin to a general medical/surgical ward. Best practice alarm recommendations that clinicians are familiar with are currently tailored to hospital settings. These may be too sensitive for less controlled environments, such as patients at home, where there may be greater activity, and clinical confirmation of deterioration is more laborious. Alarm metrics generated from four vital sign observation rates and four aggregation windows demonstrated the tradeoffs associated with each condition. These comparisons, derived from simulated scenarios, provide examples to enhance the understanding of how alarms are affected by alarm ruleset settings in a real dataset.

Remote care relies on monitoring a patient’s overall status with limited to no subjective information, such as clinical observation, placing more weight on objective information, such as vital signs and reporting systems, to detect changes in physiological status. Continuous vital signs offer a more robust picture of a patient’s overall status for clinical decision making [28] and enable early detection of changes in physiological status. Alarms based on vital signs observed at 15 min (*VS_15_*) occurred 3.25 h, 3.75 h, and 2.75 h earlier than at 4 h (*VS_4_*) for *A_1_*, *A_2_*, and *A_3_*, respectively.

A byproduct of continuously collected data is an increased number of alarms. Alarm metrics (total *W_AP_*, patient rate, and alarm rate) increased with an increased observation rate in all alarm rulesets. Two factors that may contribute to increased alarms are the conditions under which vital signs are collected and the assessment rate. A patient is generally awake, seated upright, and still during manual observation, which provides a controlled condition for vital sign measurements. Similarly, vital sign measurements collected by wearables, such as finger pulse oximeters, which are infrequent and short in duration (“spot checks”), provide snapshots of a patient in a constant state. In contrast, when a more robust picture of a patient’s status is captured through continuous data collection, it includes vital signs of a patient in different positions (lying, seated, or standing) and during a range of daily activities (sleeping, watching television, talking, walking, etc.). Variation in vital sign values, and noise, are inherent in a vital sign dataset collected in free-living conditions. In addition to the vital sign measurement conditions, the vital sign observation rate is directly related to the number of data points included in an assessment, and as the observation rate increases, the likelihood that a change in physiological status has the potential to be detected increases [3,10]. For example, vital sign observations collected every 12 h can only generate a maximum of 2 alarms/day whereas vital sign observations collected every hour can generate a maximum of 24 alarms/day. The percent of assessment windows with a positive alarm (*W_AP_*) increased by 50.9% in *A_1_*, 70.7% in *A_2_*, and 19.8% in *A_3_* from the 12-h observation rate (*VS_12_*) to the 15-min observation rate (*VS_15_*). This overload of data and a higher likelihood of triggering an alarm can be more of a burden than an asset unless alarms are designed to translate the increased vital sign data into actionable information.

Current alarm recommendations were designed for use in a hospital setting. Even so, a systematic review of vital sign alarms in the hospital setting reported that 74–80% of alarms were not actionable, and a significant relationship exists between alarm exposure and response time [22]. Direct translation of current recommendations to the remote setting, where more vital sign data are assessed and assessment is conducted under free-living conditions, will likely yield a greater number of nonactionable alarms and an increased risk of alarm fatigue [29]. Healthcare systems will need to staff their programs proportionally to support alarm response, where contact with patients for follow-up may be more challenging than in person. Alarm sensitivity, and its implications can be mitigated by a deeper understanding of how alarm parameters change alarm outcomes so that appropriate choices can be made for this new monitoring environment.

The vital signs that triggered alarms most frequently were respiratory rate, followed by SpO_2_; consistent with previous work [18]. Although respiratory rate was triggered more frequently than SpO_2_, it has been shown that pulse oximetry alarms are the largest contributor to the number of false alarms [30]. Three adjustable alarm settings to improve alarms are vital sign threshold, aggregation window, and combination rules. There was a 33% reduction in the number of assessment windows with a positive alarm when the SpO_2_ threshold was lowered from 92% to 91%, which was similar to reductions observed by reducing SpO_2_ threshold from 90% to 88% (45% reduction) [31] and 90% to 85% (61% reduction) [22]. When SpO_2_ was part of a combination rule, it was triggered less frequently (*A_3_-02*, *A_3_-03*, *A_1_-06*, *A_1_-07*, *A_1_-08*, *A_1_-09*, *A_1_-10*) (Table S1). Similarly, an aggregation window that increased from 5 min (*VS_SD_*) to 4 h (*AW_4_*) in *A_2_*—hypoxia reduced the number of assessment windows with a positive alarm by 70.6%.

The comparison between alarm rulesets illustrates that a modification to any one of the settings can influence the alarm, but there is a compound effect when two or more settings are changed. The alarm ruleset *A_3_* included one individual rule and three combination rules while *A_2_* included five individual rules. There was a 69.7–90.6% decrease in assessment windows with a positive alarm when using *A_3_* compared to *A_2_*. The subset of assessment windows with a positive alarm triggered by PR was reduced by a range of 95.7–98.7% when the PR threshold was lowered from 60 beats/min in *A_2_* to 45 beats/min in *A_3_*. The number of assessment windows with a positive alarm for *A_3_* decreased with an increase in the aggregation window. Previous work showed that tailoring both aggregation windows and vital sign thresholds produced the greatest reduction in alarms compared to tailoring either aggregation window or vital sign threshold alone [31].

Alarm rate for all conditions evaluated was less than half of the acceptable ‘upper limit’ of 10.8 alarms/day per patient reported by Prgomet et al. when continuous monitoring was implemented on a general ward [32]. The tradeoff of a reduced number of alarms is missed or late detection of deterioration. Alarm metrics (*W_AP_*, patient rate, and alarm rate) decreased with increased aggregation windows in all alarm rulesets, without decreasing *EDT*. There are two elements to include in the interpretation of the *EDT* comparison between aggregation windows to understand why a larger aggregation window may have resulted in an earlier detection time. The first is that the number of *W_AP_* included in the median *EDT* calculation was greater for smaller aggregation windows. For example, there were 4-h assessment windows where a trigger occurred in *AW_15_* with a low *EDT* but was not triggered in *AW_1_*. The median *EDT* in *AW_15_* might have been lowered by the additional *W_AP_* but remained high for *AW_1_*. The second is the number of vital sign observations included in the aggregation window. The smaller the aggregation window, the faster the data points outside of the ‘normal range’ were removed from the median calculation. For example, the median vital signs in *AW_1_* and *AW_4_* were smoothed over more time, potentially causing the vital sign to stay steady under the alarm rule threshold, while smaller aggregation windows (*AW_15_* and *VS_SD_*) may have fluctuated above and below the threshold value (Figure S1). The consequence of this was a carryover effect between consecutive assessment windows, which resulted in an alarm at the beginning of the second assessment window. There was a higher chance of carryover with a larger aggregation window because, as described above, larger aggregation windows included more vital sign observations in the median calculation.

Another factor to consider is what happens at the patient level when deciding on combination vs. individual alarm rules, aggregation window size, and/or vital sign threshold. Average alarms per day generated by one rule were used to illustrate the effects of these clinical decisions. Patient 51, for example, was highly adherent to wearing the CH wearable (91.0%), and the patient’s pulse rate and respiratory rate were in the normal ranges (PR = 80.1 (9.2) beats/min; and RR = 21.9 (2.4) breaths/min). However, this patient presented with low oxygen saturation (SpO_2_ = 92.6 (3.4)%) during admission (Figure 2). The assumption in this example was that the vital sign observation rate was not a modifiable factor and was set to the CH wearable vital sign observation rate. Let’s say that the clinician decided to use a combination rule: SpO_2_ < 90% and RR > 25 breaths/min. The average alarms per day would change based on aggregation window size: *VS_SD_* = 0.9 ± 0.9 alarms/day, *AW_15_* = 0.6 ± 0.9 alarms/day, *AW_1_* = 0.2 ± 0.6 alarms/day, *AW_4_* = 0.1 ± 0.3 alarms/day. In other words, if Patient 51 had an *LoS* = 10 days, using *VS_SD_* would result in a follow-up call by the clinician almost 10 times during their *LoS,* whereas using *AW_1_* would result in a follow-up call approximately twice during their *LoS*. Now, let’s say that the clinician decided to use an individual rule (SpO_2_ = 91%) and *AW_1_*. Patient 51 would generate 2.3 ± 1.9 alarms/day. Alarms per day would increase even more to 3.1 ± 2.0 alarms/day had the clinician selected a higher vital sign threshold (SpO_2_ = 92%).

The vital sign observations of Patient 51 during CH admission were slightly higher for PR and RR, and slightly lower for SpO_2_, compared to the median vital sign observations at the group level (PR = 72.9 (19.4) beats/min, RR = 19.3 (6.4) breaths/min, and SpO_2_ = 95.4 (3.6)%). Average alarms per day were similarly higher than those reported at the group level (Table S1). There were scenarios, however, where the average alarms per day were lower than expected. During CH admission, the vital sign observations for Patient 58 were PR = 59.2 (4.0) beats/min, RR = 16.8 (1.4) breaths/min, and SpO_2_ = 79.2 (0.6)%. This patient had lower observation values compared to the group level for all vital signs. While both patients had low oxygen saturation, Patient 58 SpO_2_ was much lower than Patient 51. When the same clinical decisions outlined for patient 51 were applied to the data for patient 58, it was hypothesized that a median SpO_2_ as low as 79.2% would generate high average alarms per day, especially for individual SpO_2_ rules. However, Patient 58 generated zero alarms for the combination rule (SpO_2_ < 90% and RR > 25 breaths/min) for all aggregation window sizes. When either of the individual rules (SpO_2_ = 91% or SpO_2_ = 92%) was paired with *AW_1_*, zero alarms were generated. It was only when using an extremely sensitive setting (individual rules with *AW_15_* and *VS_SD_*) that alarms were generated for Patient 58 (*AW_15_* = 0.4 alarms/day, *VS_SD_* = 0.6 alarms/day). The reason for the lack of alarms was because of patient 58’s low adherence (19.9%) to wearing the CH wearable. While the patient’s extremely low SpO_2_ is likely a result of improper equipment use, the value served the purpose of this example to demonstrate that vital sign observation data need to exist to generate alarms, and sounds a cautionary note that rules based on population data may overlook vulnerable individuals.

Remote monitoring is predicated on data flowing from patient to provider, which is only possible when patients actually use the monitoring equipment as prescribed. The biggest factor that impacts the data is patient adherence to wearing/using the equipment as prescribed. The median adherence of the healthcare at home dataset was 64.6%, indicating that patients wore the Current Health wearable more than half of the time during their admission. Patterns in wear habits can be extracted from the metadata and used to time nudges to potentially increase adherence. The Current Health platform includes alarms for missing data as a prompt to the clinician so they can reach out to the patient, another technique to improve adherence. Other factors to address when deploying remote patient monitoring equipment are education to support proper use of the equipment, especially if body placement is important, a contingency plan in the event of failed data transmission, and how to account for movement when the ideal condition to collect vital sign measurements is at rest.

The conclusions that could be drawn from this work were limited, as the vital sign dataset was not labeled with clinical outcomes. If it were, we could have differentiated those vital sign changes that corresponded to genuine clinical deterioration rather than physiological variability or artifacts. Without this information, we can only describe the principles by which alarm decisions can be made, and the likely impact of changes on alarm volume. This alludes to a broader problem in which vendors typically design monitoring products without the ‘feedback loop’ of clinical utility. Monitoring products are developed, integrated into the clinical workflow, and used by healthcare providers, and only then can real-world feedback be sought on their alarm parameters.

Consequently, assumptions are made, and proxies are created that enable vendors to make use of unlabeled data to develop their products and create default settings. For example, one method trialed to create a reference for this study was to use NEWS2, an objective measure used as part of the standard of care, and an observation rate of 4 h, common in the acute care setting [9]. Labels were created as a proxy of physiologic values that warranted follow-up based on the subset of rules from the NEWS2 ruleset tied to clinical escalation recommendations (scores of 3 or higher for a single vital sign or a 5 or higher for a combination of vital signs). This condition alarmed 20.9% of the assessment windows. There was at least 1 alarm in 85% of the patients, with approximately 1 alarm/day per patient. However, a 4-h observation rate may not be suitable for remote monitoring. Although 15 conditions produced more assessment windows with positive alarms than this condition, these may be a more accurate reflection of a patient’s change in status. Only 5 of these conditions had an alarm rate per patient above 3.5 alarms/day. These alarm rates are lower than the reported in-hospital and other at-home alarm rates per patient of 10.8 alarms/day [32] and 3.42 alarms/day [33]. Thus, while this proxy may have been a good representation of alarms that would necessitate clinical follow-up and a ‘sense check’ for our findings, there was still no way to verify it without feedback from the clinician. While trigger-based alarms and systematic evaluations of the impact of alarm settings are a strong foundation for physiological track and trigger systems, the information needed to develop more sophisticated algorithms that are optimized for specificity requires engagement from clinicians. The clinical utility of alarms is critical to making them more effective, and outcomes need to be fed into the system by the user (healthcare providers). Differentiation between a true alarm, a true and clinically significant alarm, and a false alarm enables the development of alarm algorithms using more sophisticated approaches, such as machine learning algorithms [34], that provide greater specificity without compromising sensitivity, which minimizes (if not eliminates) the clinical risk of caring for patients in the home.

## 5. Conclusions

Evaluation of how well standard tools, practice, and clinical interpretation translate to a remote setting is needed as healthcare transitions into the home. It should not be assumed that they will be equally effective in this comparatively new environment. Total alarms ranged between conditions of vital sign observation frequency and/or alarm setting, from 65 to 3113. Vital sign thresholds closest to the normal range, smaller aggregation window, and/or individual vital sign rule were the conditions most likely to increase alarm rates and were therefore most amenable to modification to increase specificity and minimize the potential for alarm fatigue. Although technology is meant to enable clinicians, the majority of innovations live as feedforward systems, but clinical utility is the foundation of alarm optimization. There is a need to shift from the current feedforward dynamic between vendor/healthcare provider to one of a feedback loop to further develop products that best support the healthcare system.

## Figures and Tables

**Table 1:** Alarm rulesets.

Alarm Ruleset	Alarm Rule	Respiratory Rate	Oxygen Saturation	Pulse Rate
*A_1_*	*A_1_-01*	RR < 9		
	*A_1_-02*	RR > 24		
	*A_1_-03*		SpO_2_ ≤ 91	
	*A_1_-04*			PR < 41
	*A_1_-05*			PR > 130
	*A_1_-06* *	9 ≤ RR ≤ 11	92 ≤ SpO_2_ ≤ 93	111 ≤ PR ≤ 130
	*A_1_-07* *	21 ≤ RR ≤ 24	92 ≤ SpO_2_ ≤ 93	91 ≤ PR ≤ 110
	*A_1_-08* *	21 ≤ RR ≤ 24	94 ≤ SpO_2_ ≤ 95	111 ≤ PR ≤ 130
	*A_1_-09* *	21 ≤ RR ≤ 24	92 ≤ SpO_2_ ≤ 93	111 ≤ PR ≤ 130
	*A_1_-10* *	21 ≤ RR ≤ 24	92 ≤ SpO_2_ ≤ 93	41 ≤ PR ≤ 50
*A_2_*	*A_2_-01* (Hypoxia)		SpO_2_ < 92	
	*A_2_-02* (Tachycardia)			PR > 100
	*A_2_-03* (Bradycardia)			PR < 60
	*A_2_-04* (Tachypnea)	RR > 20		
	*A_2_-05* (Bradypnea)	RR < 10		
*A_3_*	A_3_-01 *	RR > 25		PR > 90
	A_3_-02 *	RR > 25	SpO_2_ < 90	
	A_3_-03 *	RR < 10	SpO_2_ < 90	
	*A_3_-04*			PR < 45

* Combination rule.

**Table 2:** Alarm metrics.

Ruleset	Observation Rate	Aggregation Window	*W_AP_*	Patient Rate (%)	Alarm Rate	EDT (hour)
*A_1_*	*VS_12_*	*AW_0_*	235	64.47	0.33 ± 0.56	0 (0)
	*VS_4_*	*AW_0_*	683	85.53	0.96 ± 1.37	0 (0)
	*VS_1_*	*AW_0_*	1364	94.74	1.93 ± 1.83	3 (2.00)
	*VS_15_*	*AW_0_*	1899	98.68	2.68 ± 1.99	3.25 (1.75)
	*VS_SD_*	-	2251	100	3.18 ± 2.02	3.5 (1.52)
	*VS_OD_*	*AW_15_*	1759	96.05	2.48 ± 2.02	3.41 (1.76)
	*VS_OD_*	*AW_1_*	1195	76.32	1.69 ± 1.88	3.67 (1.68)
	*VS_OD_*	*AW_4_*	791	65.79	1.12 ± 1.65	3.97 (1.45)
*A_2_*	*VS_12_*	*AW_0_*	609	93.42	0.86 ± 0.76	0 (0)
	*VS_4_*	*AW_0_*	1737	96.05	2.45 ± 1.89	0 (0)
	*VS_1_*	*AW_0_*	2548	98.68	3.60 ± 1.92	3 (1.00)
	*VS_15_*	*AW_0_*	2922	100	4.13 ± 1.78	3.75 (0.75)
	*VS_SD_*	-	3113	100	4.40 ± 1.68	3.96 (0.55)
	*VS_OD_*	*AW_15_*	2855	100	4.03 ± 1.83	3.98 (0.74)
	*VS_OD_*	*AW_1_*	2467	98.68	3.48 ± 2.04	4.00 (0.79)
	*VS_OD_*	*AW_4_*	2121	93.42	3.00 ± 2.22	4.00 (0.28)
*A_3_*	*VS_12_*	*AW_0_*	65	30.26	0.09 ± 0.31	0 (0)
	*VS_4_*	*AW_0_*	185	50.00	0.26 ± 0.71	0 (0)
	*VS_1_*	*AW_0_*	435	61.84	0.61 ± 1.16	2.00 (2.00)
	*VS_15_*	*AW_0_*	712	65.79	1.01 ± 1.50	2.75 (2.00)
	*VS_SD_*	-	942	80.26	1.33 ± 1.66	3.05 (2.05)
	*VS_OD_*	*AW_15_*	626	65.79	0.88 ± 1.45	3.04 (2.16)
	*VS_OD_*	*AW_1_*	356	48.68	0.50 ± 1.13	3.25 (2.16)
	*VS_OD_*	*AW_4_*	200	32.89	0.28 ± 0.88	3.84 (1.90)

## Data Availability

The data presented in this study are available on request from the corresponding author.

## Supplementary Materials

The following supporting information can be downloaded at: https://www.mdpi.com/article/10.3390/bioengineering10010037/s1, Figure S1: An example of vital sign data at different observation rates and aggregation windows (*AW*) during a 4-h assessment window in one patient. Figure S2: Diagram of the data processing and alarm simulator. Table S1: Alarm rule metrics.

## Appendix A

**Table A1:** Minimum number of observations for each aggregation window (*AW*).

	Minimum Observations		
*AW*	PR	SpO_2_	RR
5 min	30	30	15
15 min	90	90	45
1 h	360	360	180
4 h	1440	1440	720
