# Supplementary material for: Assessment of Remote Vital Sign Monitoring and Alarms in a Real-World Healthcare at Home Dataset
Source: Bioengineering (Basel). 2022 Dec 28;10(1):37. doi: 10.3390/bioengineering10010037 (PMC9854741; doi:10.3390/bioengineering10010037)
Supplement: Supplementary file 1 [file bioengineering-10-00037-s001.zip › Supplemental Material/Figure S1.pdf]

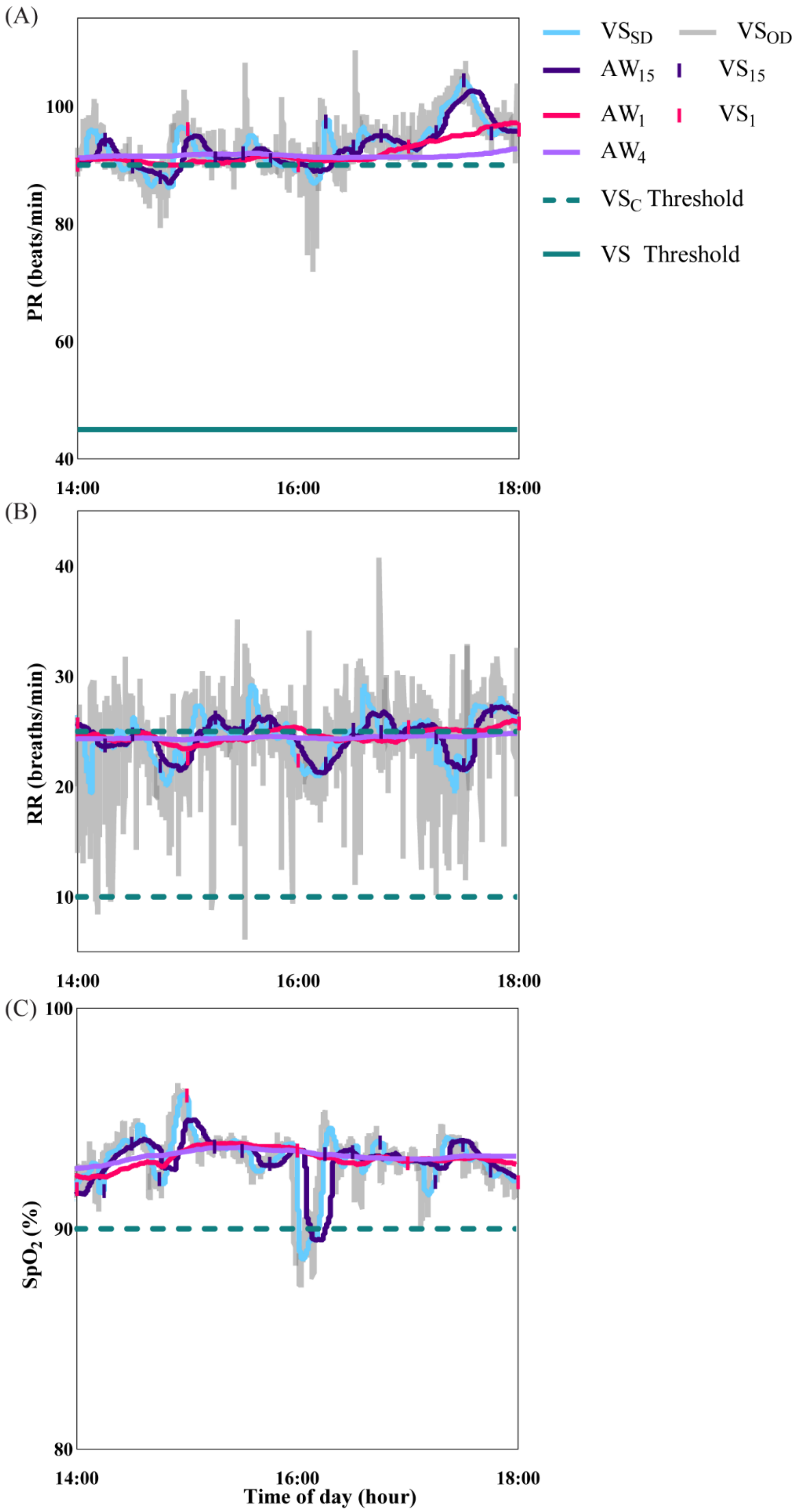
(A)
PR (beats/min)
100
80
60
40
14:00
16:00
18:00
VS_{SD}
VS_{OD}
AW_{15}
VS_{15}
AW_1
VS_1
AW_4
VS_C Threshold
VS Threshold
(B)
RR (breaths/min)
40
30
20
10
14:00
16:00
18:00
(C)
SpO_2 (%)
100
90
80
14:00
16:00
18:00
Time of day (hour)

Figure S1. An example of vital sign data at different observation rates and aggregation windows (*AW*) during a 4-hour assessment window in one patient. (A) Pulse rate (PR); (B) Respiratory rate (RR); (C) Oxygen saturation (SpO_2). The vital sign threshold values in this example were selected from A_3. Solid horizontal lines are thresholds used in single vital sign rules (VS Threshold) and dashed horizontal lines are thresholds used in combination rules (VS_C Threshold). VS_{OD} = original dataset; VS_{SD} = 5-minute aggregation window; VS_{15} = 15-minute observation rate; VS_1 = 1-hour observation rate; AW_{15} = 15-minute aggregation window; AW_1 = 1-hour aggregation window; AW_4 = 4-hour aggregation window.

The variability in the original vital sign dataset was reduced with a 5-minute aggregation window (VS_{SD}). An example of the difference in vital sign value between smoothed (aggregation window) vs. downsampled data was observed in the SpO_2 values of VS_1 and AW_1 at approximately 15:00 (panel C). This difference demonstrates one benefit of incorporating the last hour of vital sign observations (AW_1). In VS_1, the SpO_2 value at 15:00 was ~5% higher than at 14:00 and 16:00. However, vital sign data smoothed with longer aggregation windows took longer to react to temporary changes in the trend. This was apparent in PR (panel A) and RR (panel C). Pulse rate for AW_4 remained above VS_C Threshold (PR = 90 beats/min) during the 4-hour assessment window whereas PR for other conditions fluctuated above and below the threshold. Respiratory rate for AW_4 remained below the VS_C Threshold during the 4-hour assessment window while other conditions breached the threshold. An alarm was not triggered for AW_4 during this assessment window.
