# Supplementary material for: Assessment of Remote Vital Sign Monitoring and Alarms in a Real-World Healthcare at Home Dataset
Source: Bioengineering (Basel). 2022 Dec 28;10(1):37. doi: 10.3390/bioengineering10010037 (PMC9854741; doi:10.3390/bioengineering10010037)
Supplement: Supplementary file 1 [file bioengineering-10-00037-s001.zip › Supplemental Material/Figure S2.pdf]

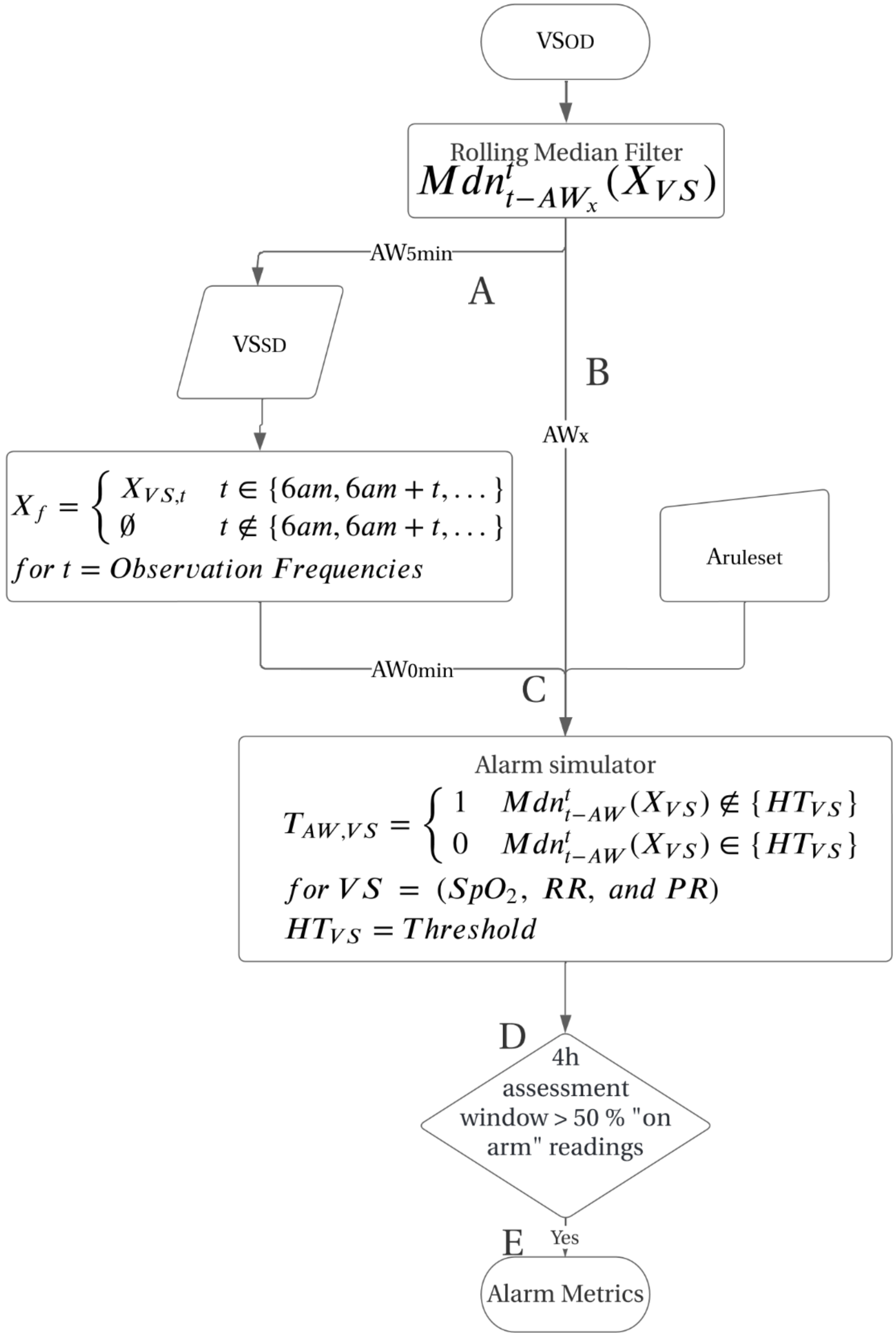

Figure S2. Diagram of data processing and alarm simulator: test conditions were created with the vital sign observation dataset (VS_{OD}) by (A) filtering with a 5-minute aggregation window (AW_{5min}) and downsampling, or (B) passing VS_{OD} through an alarm simulator with different aggregation windows (AW_x) applied to the rolling median filter. *Mdn* = Median. (A) VS_{SD} was created with a 5-minute rolling median filter (AW_{5min}) and then downsampled to 4 vital sign observation frequencies. t = 15min, 1hr, 4hr, 12hr. X_{VS} = Pulse Rate, Respiratory Rate, or

Oxygen Saturation array. Ø = no data. (B) VS_{OD} was passed through the rolling median filter with different sized aggregation windows (AW_x). AW_x = 5min (VS_{SD}), 15min, 1hr, 4hr. (C) Test conditions were passed through the alarm simulator with defined rulesets outlined in Table 1 ($A_{ruleset}$). Downsampled data passed through the alarm simulator with AW_x = 0 min. Triggers ($T_{AW,VS}$) were created for each condition. For combination rules, triggers (T_{AW}) for each AW were created by summarizing across the $T_{AW,VS}$ for the vital signs included in the combination rule: T_{AW} are equal to 1 when all the $T_{AW,VS}$ in the rule are equal to 1 within 30s of each other. (D) A patient's length of stay was divided into 4-hour windows. 4-hour windows with > 50% adherence were included in alarm assessment. (E) Alarm triggers ($T_{AW,VS}$) were grouped into the 4-hour assessment windows and counted as a positive alarm window (W_{AP}). The alarm metrics alarm rate, patient rate, and early detection time (EDT) were calculated based on W_{AP}.
