# Supplementary material for: Assessment of Remote Vital Sign Monitoring and Alarms in a Real-World Healthcare at Home Dataset
Source: Bioengineering (Basel). 2022 Dec 28;10(1):37. doi: 10.3390/bioengineering10010037 (PMC9854741; doi:10.3390/bioengineering10010037)
Supplement: Supplementary file 1 [file bioengineering-10-00037-s001.zip › Supplemental Material/Table S1.pdf]

Table S1. Alarm rule metrics

Ruleset	Alarm	Observation Rate	Aggregation Window	W_{AP}	Patient Rate (%)	Alarm Rate
A_1	A_1 - 01	VS_{12}	AW_0	1	1.32	0 ± 0.04
	A_1 - 02	VS_{12}	AW_0	157	44.74	0.22 ± 0.47
	A_1 - 03	VS_{12}	AW_0	97	44.74	0.14 ± 0.38
	A_1 - 04	VS_{12}	AW_0	0	0	0 ± 0
	A_1 - 05	VS_{12}	AW_0	1	1.32	0 ± 0.04
	A_1 - 06	VS_{12}	AW_0	0	0	0 ± 0
	A_1 - 07	VS_{12}	AW_0	3	3.95	0 ± 0.07
	A_1 - 08	VS_{12}	AW_0	1	1.32	0 ± 0.04
	A_1 - 09	VS_{12}	AW_0	0	0	0 ± 0
	A_1 - 10	VS_{12}	AW_0	0	0	0 ± 0
A_2	A_2 - 01	VS_{12}	AW_0	133	50	0.19 ± 0.45
	A_2 - 02	VS_{12}	AW_0	82	21.05	0.12 ± 0.36
	A_2 - 03	VS_{12}	AW_0	107	36.84	0.15 ± 0.39
	A_2 - 04	VS_{12}	AW_0	446	82.89	0.63 ± 0.73
	A_2 - 05	VS_{12}	AW_0	3	3.95	0 ± 0.07
A_3	A_3 - 01	VS_{12}	AW_0	53	19.74	0.07 ± 0.29
	A_3 - 02	VS_{12}	AW_0	15	15.79	0.02 ± 0.14
	A_3 - 03	VS_{12}	AW_0	0	0	0 ± 0
	A_3 - 04	VS_{12}	AW_0	2	2.63	0 ± 0.05
A_1	A_1 - 01	VS_4	AW_0	1	1.32	0 ± 0.04
	A_1 - 02	VS_4	AW_0	463	64.47	0.65 ± 1.14
	A_1 - 03	VS_4	AW_0	273	61.84	0.39 ± 0.86
	A_1 - 04	VS_4	AW_0	0	0	0 ± 0
	A_1 - 05	VS_4	AW_0	3	3.95	0 ± 0.07
	A_1 - 06	VS_4	AW_0	0	0	0 ± 0
	A_1 - 07	VS_4	AW_0	10	6.58	0.01 ± 0.12
	A_1 - 08	VS_4	AW_0	1	1.32	0 ± 0.04
	A_1 - 09	VS_4	AW_0	1	1.32	0 ± 0.04
	A_1 - 10	VS_4	AW_0	0	0	0 ± 0
A_2	A_2 - 01	VS_4	AW_0	378	71.05	0.53 ± 1.00
	A_2 - 02	VS_4	AW_0	260	38.16	0.37 ± 0.94
	A_2 - 03	VS_4	AW_0	249	44.74	0.35 ± 0.84
	A_2 - 04	VS_4	AW_0	1275	93.42	1.80 ± 1.80
	A_2 - 05	VS_4	AW_0	6	6.58	0.01 ± 0.09
A_3	A_3 - 01	VS_4	AW_0	149	36.84	0.21 ± 0.65
	A_3 - 02	VS_4	AW_0	49	25	0.07 ± 0.33
	A_3 - 03	VS_4	AW_0	0	0	0 ± 0
	A_3 - 04	VS_4	AW_0	5	3.95	0.01 ± 0.08
A_1	A_1 - 01	VS_1	AW_0	2	2.63	0 ± 0.05

	A_1 - 02	VS_1	AW_0	973	82.89	1.37 ± 1.65
	A_1 - 03	VS_1	AW_0	663	77.63	0.94 ± 1.39
	A_1 - 04	VS_1	AW_0	2	1.32	0 ± 0.08
	A_1 - 05	VS_1	AW_0	7	6.58	0.01 ± 0.11
	A_1 - 06	VS_1	AW_0	0	0	0 ± 0
	A_1 - 07	VS_1	AW_0	18	13.16	0.03 ± 0.16
	A_1 - 08	VS_1	AW_0	2	2.63	0 ± 0.05
	A_1 - 09	VS_1	AW_0	2	1.32	0 ± 0.08
	A_1 - 10	VS_1	AW_0	0	0	0 ± 0
A_2	A_2 - 01	VS_1	AW_0	863	88.16	1.22 ± 1.59
	A_2 - 02	VS_1	AW_0	461	52.63	0.65 ± 1.30
	A_2 - 03	VS_1	AW_0	500	57.89	0.71 ± 1.33
	A_2 - 04	VS_1	AW_0	2060	98.68	2.91 ± 2.11
	A_2 - 05	VS_1	AW_0	33	17.11	0.05 ± 0.24
A_3	A_3 - 01	VS_1	AW_0	331	51.32	0.47 ± 1.04
	A_3 - 02	VS_1	AW_0	144	34.21	0.2 ± 0.64
	A_3 - 03	VS_1	AW_0	1	1.32	0 ± 0.04
	A_3 - 04	VS_1	AW_0	15	5.26	0.02 ± 0.23
A_1	A_1 - 01	VS_{15}	AW_0	4	5.26	0.01 ± 0.08
	A_1 - 02	VS_{15}	AW_0	1428	90.79	2.01 ± 1.92
	A_1 - 03	VS_{15}	AW_0	1017	84.21	1.43 ± 1.70
	A_1 - 04	VS_{15}	AW_0	4	3.95	0.01 ± 0.09
	A_1 - 05	VS_{15}	AW_0	14	11.84	0.02 ± 0.14
	A_1 - 06	VS_{15}	AW_0	0	0	0 ± 0
	A_1 - 07	VS_{15}	AW_0	47	19.74	0.07 ± 0.30
	A_1 - 08	VS_{15}	AW_0	9	5.26	0.01 ± 0.15
	A_1 - 09	VS_{15}	AW_0	3	1.32	0 ± 0.08
	A_1 - 10	VS_{15}	AW_0	0	0	0 ± 0
A_2	A_2 - 01	VS_{15}	AW_0	1261	92.11	1.78 ± 1.83
	A_2 - 02	VS_{15}	AW_0	627	63.16	0.88 ± 1.51
	A_2 - 03	VS_{15}	AW_0	652	59.21	0.92 ± 1.54
	A_2 - 04	VS_{15}	AW_0	2533	100	3.57 ± 1.97
	A_2 - 05	VS_{15}	AW_0	75	25	0.11 ± 0.40
A_3	A_3 - 01	VS_{15}	AW_0	525	57.89	0.74 ± 1.35
	A_3 - 02	VS_{15}	AW_0	292	42.11	0.41 ± 1.00
	A_3 - 03	VS_{15}	AW_0	3	3.95	0 ± 0.07
	A_3 - 04	VS_{15}	AW_0	22	10.53	0.03 ± 0.25
A_1	A_1 - 01	VS_{SD}	-	12	11.84	0.02 ± 0.14
	A_1 - 02	VS_{SD}	-	1849	97.37	2.61 ± 2.01
	A_1 - 03	VS_{SD}	-	1223	88.16	1.72 ± 1.83
	A_1 - 04	VS_{SD}	-	7	5.26	0.01 ± 0.15
	A_1 - 05	VS_{SD}	-	21	15.79	0.03 ± 0.18
	A_1 - 06	VS_{SD}	-	0	0	0 ± 0

	A_1 - 07	VS_{SD}	-	181	31.58	0.26 ± 0.71
	A_1 - 08	VS_{SD}	-	30	11.84	0.04 ± 0.23
	A_1 - 09	VS_{SD}	-	15	9.21	0.02 ± 0.15
	A_1 - 10	VS_{SD}	-	4	2.63	0.01 ± 0.08
A_2	A_2 - 01	VS_{SD}	-	1501	94.74	2.12 ± 1.95
	A_2 - 02	VS_{SD}	-	722	64.47	1.02 ± 1.61
	A_2 - 03	VS_{SD}	-	782	63.16	1.1 ± 1.69
	A_2 - 04	VS_{SD}	-	2859	100	4.03 ± 1.83
	A_2 - 05	VS_{SD}	-	223	59.21	0.31 ± 0.76
A_3	A_3 - 01	VS_{SD}	-	697	63.16	0.98 ± 1.54
	A_3 - 02	VS_{SD}	-	430	60.53	0.61 ± 1.22
	A_3 - 03	VS_{SD}	-	3	2.63	0 ± 0.07
	A_3 - 04	VS_{SD}	-	34	15.79	0.05 ± 0.31
A_1	A_1 - 01	VS_{OD}	AW_{15}	0	0	0 ± 0
	A_1 - 02	VS_{OD}	AW_{15}	1324	88.16	1.87 ± 1.94
	A_1 - 03	VS_{OD}	AW_{15}	877	81.58	1.24 ± 1.59
	A_1 - 04	VS_{OD}	AW_{15}	4	3.95	0.01 ± 0.09
	A_1 - 05	VS_{OD}	AW_{15}	4	5.26	0.01 ± 0.08
	A_1 - 06	VS_{OD}	AW_{15}	0	0	0 ± 0
	A_1 - 07	VS_{OD}	AW_{15}	88	21.05	0.12 ± 0.46
	A_1 - 08	VS_{OD}	AW_{15}	14	7.89	0.02 ± 0.18
	A_1 - 09	VS_{OD}	AW_{15}	4	3.95	0.01 ± 0.08
	A_1 - 10	VS_{OD}	AW_{15}	0	0	0 ± 0
A_2	A_2 - 01	VS_{OD}	AW_{15}	1107	84.21	1.56 ± 1.76
	A_2 - 02	VS_{OD}	AW_{15}	537	51.32	0.76 ± 1.45
	A_2 - 03	VS_{OD}	AW_{15}	641	60.53	0.90 ± 1.53
	A_2 - 04	VS_{OD}	AW_{15}	2460	100	3.47 ± 2.05
	A_2 - 05	VS_{OD}	AW_{15}	32	14.47	0.05 ± 0.27
A_3	A_3 - 01	VS_{OD}	AW_{15}	471	53.95	0.66 ± 1.31
	A_3 - 02	VS_{OD}	AW_{15}	254	43.42	0.36 ± 0.96
	A_3 - 03	VS_{OD}	AW_{15}	0	0	0 ± 0
	A_3 - 04	VS_{OD}	AW_{15}	18	9.21	0.03 ± 0.21
A_1	A_1 - 01	VS_{OD}	AW_1	0	0	0 ± 0
	A_1 - 02	VS_{OD}	AW_1	873	59.21	1.23 ± 1.71
	A_1 - 03	VS_{OD}	AW_1	516	56.58	0.73 ± 1.32
	A_1 - 04	VS_{OD}	AW_1	0	0	0 ± 0
	A_1 - 05	VS_{OD}	AW_1	2	2.63	0 ± 0
	A_1 - 06	VS_{OD}	AW_1	0	0	0 ± 0
	A_1 - 07	VS_{OD}	AW_1	35	14.47	0.05 ± 0.25
	A_1 - 08	VS_{OD}	AW_1	2	2.63	0 ± 0.05
	A_1 - 09	VS_{OD}	AW_1	2	2.63	0 ± 0.05
	A_1 - 10	VS_{OD}	AW_1	0	0	0 ± 0
A_2	A_2 - 01	VS_{OD}	AW_1	701	67.11	0.99 ± 1.52

	A_2 - 02	VS_{OD}	AW_1	369	38.16	0.52 ± 1.24
	A_2 - 03	VS_{OD}	AW_1	501	53.95	0.71 ± 1.41
	A_2 - 04	VS_{OD}	AW_1	2011	93.42	2.84 ± 2.21
	A_2 - 05	VS_{OD}	AW_1	6	3.95	0.01 ± 0.14
A_3	A_3 - 01	VS_{OD}	AW_1	284	39.47	0.4 ± 1.04
	A_3 - 02	VS_{OD}	AW_1	107	26.32	0.15 ± 0.58
	A_3 - 03	VS_{OD}	AW_1	0	0	0 ± 0
	A_3 - 04	VS_{OD}	AW_1	9	3.95	0.01 ± 0.15
A_1	A_1 - 01	VS_{OD}	AW_4	0	0	0 ± 0
	A_1 - 02	VS_{OD}	AW_4	563	46.05	0.79 ± 1.42
	A_1 - 03	VS_{OD}	AW_4	306	42.11	0.43 ± 1.08
	A_1 - 04	VS_{OD}	AW_4	0	0	0 ± 0
	A_1 - 05	VS_{OD}	AW_4	0	0	0 ± 0
	A_1 - 06	VS_{OD}	AW_4	0	0	0 ± 0
	A_1 - 07	VS_{OD}	AW_4	13	7.89	0.02 ± 0.14
	A_1 - 08	VS_{OD}	AW_4	0	0	0 ± 0
	A_1 - 09	VS_{OD}	AW_4	1	1.32	0 ± 0.04
	A_1 - 10	VS_{OD}	AW_4	0	0	0 ± 0
A_2	A_2 - 01	VS_{OD}	AW_4	441	51.32	0.62 ± 1.29
	A_2 - 02	VS_{OD}	AW_4	255	26.32	0.36 ± 1.05
	A_2 - 03	VS_{OD}	AW_4	393	42.11	0.55 ± 1.30
	A_2 - 04	VS_{OD}	AW_4	1685	84.21	2.38 ± 2.25
	A_2 - 05	VS_{OD}	AW_4	3	1.32	0 ± 0.11
A_3	A_3 - 01	VS_{OD}	AW_4	172	25	0.24 ± 0.84
	A_3 - 02	VS_{OD}	AW_4	46	14.47	0.06 ± 0.37
	A_3 - 03	VS_{OD}	AW_4	0	0	0 ± 0
	A_3 - 04	VS_{OD}	AW_4	5	1.32	0.01 ± 0.12
